# Mysteries of Type I IFN Response: Benefits Versus Detriments

**DOI:** 10.3389/fimmu.2015.00021

**Published:** 2015-01-28

**Authors:** Yoichi Furuya, Herbert P. Ludewick, Arno Müllbacher

**Affiliations:** ^1^Center for Immunology and Microbial Disease, Albany Medical College, Albany, NY, USA; ^2^Department of Immunology, The John Curtin School of Medical Research, Australian National University, Canberra, ACT, Australia

**Keywords:** type I interferons, vaccine adjuvant, viral, bacterial

Successful containment of infection is dependent on both innate and adaptive immune responses. Cytokines are essential components of both of these systems. In particular, type I interferons (IFN-I) are important components of early innate immunity against infections. However, the production of IFN-I could serve as a double-edged sword, in that it could help eliminate infections or in certain instances enhance host’s susceptibility to infections. For example, IFN-I provide early resistance against acute viral infections, but are detrimental to the host during certain bacterial infections and chronic viral infections. This Research Topic presents seven articles that address the biological roles of IFN-Is in host immunity and various contexts (e.g., autoimmunity, cancer, viral/bacterial infections, IFN-I therapy, etc.) where IFN-Is could either serve to control or exacerbate disease.

The original research performed by Babb et al. investigates the potential adjuvant activity of gamma-irradiated influenza (1). They demonstrate that co-vaccination with gamma-irradiated-influenza virus and poorly immunogenic inactivated Semliki forest virus (SFV) results in enhanced SFV-specific antibody responses without compromising humoral immunity against influenza infections. They have previously shown that gamma-irradiation destroys the ability of SFV but not influenza A virus to elicit strong IFN-I responses (2). Thus, it is likely that the adjuvant activity of gamma-irradiated influenza virus is attributable to its potency of IFN-I induction. These authors speculate that gamma-irradiated influenza virus may therefore be exploited as an adjuvant to improve the efficacy of poorly immunogenic vaccines owing to its ability to stimulate IFN-I production.

The potent immune activating properties of IFN-I can also be detrimental during viral infection, particularly for viruses that establish chronic infection. This certainly is the case for human or simian immunodeficiency virus (HIV/SIV) infection in non-natural hosts. As discussed by Tomasello et al. (3) and Furuya et al. (4), IFN-I induced during HIV infections may play a pathogenic role in driving chronic immune activation associated with CD4^+^ T cell depletion and loss of T cell function. Indeed, a strong correlation exists between chronic low level productions of IFN-I and disease progression in non-natural hosts. Thus, Furuya et al. hypothesize that regulatory mechanisms must exist in natural hosts that actively suppress IFN-I responses despite viral replication (4). It is speculated that understanding how the host can co-exist with HIV without generating an IFN-I response will be crucial in developing therapeutic interventions that can prevent or dampen progression to an acquired immune deficiency syndrome (AIDS). Perhaps, as pointed out by Tomasello et al., the more important question that needs to be addressed first is whether a causal link exists between low levels of IFN-I signaling and the development of AIDS in non-natural hosts or, rather, the up-regulation of IFN-stimulated genes simply a marker of disease progression.

The pathogenic role of IFN-I is more widely observed in bacterial infections. For example, the facultative intracellular bacterium *Francisella tularensis* is less virulent in mice deficient in IFN-I receptors (5, 6). Furuya et al. propose in an opinion article that the detrimental role of IFN-I during pulmonary tularemia may be linked to its suppressive effects on neutrophil recruitment, a response that may be protective against respiratory *Francisella tularensis* infection (7). The role of neutrophils during pulmonary tularemia has yet to be fully defined, but it is likely that both the magnitude and timing of cellular recruitment to the lung will determine whether neutrophils promote bacterial clearance or contribute to immunopathology.

In three review articles, the mechanisms that have been proposed to explain the IFN-I-mediated increases in bacterial infection susceptibility are discussed. The review article by Eshleman et al. focuses on the suppressive effects of IFN-I on myeloid cells during intracellular bacterial infections (8). These authors also emphasize that IFN-I can exert a positive anti-inflammatory effect in a number of autoimmune diseases and that this is mediated by the suppression of myeloid cell inflammatory responses. The review article from Dhariwala et al. describes multiple pathways of IFN-I-dependent cell deaths that may aid bacteria escape phagocytosis and thereby contributes to bacterial pathogenesis (9). Indeed, as the author pointed out, bacterial pathogens that benefits from IFN-I signaling are often facultative intracellular bacteria. The final review by Wijesundara et al. highlights pathological contexts in which IFN-I could be exploited in therapy and vaccine design with a particular emphasis on IFN-epsilon (10). IFN-epsilon appears to have both overlapping and distinct functions compared to IFN-alpha and -beta, and therefore, the author urge the need of evaluating contribution of the different members of IFN-I to fully exploit its beneficial effects.

Despite IFN-I being perhaps the most studied cytokine, their immunoregulatory roles are not fully understood. As is evident from the articles presented in this Research Topic, the role played by IFN-I is highly context-dependent and can be both beneficial and detrimental. Further research is clearly required in order for us to selectively harness the protective role of IFN-I while suppressing its damaging effects.

## Conflict of Interest Statement

The authors declare that the research was conducted in the absence of any commercial or financial relationships that could be construed as a potential conflict of interest.
